# Influence of surface characteristics of implant materials on MRSA biofilm formation and effects of antimicrobial treatment

**DOI:** 10.3389/fmicb.2023.1145210

**Published:** 2023-04-20

**Authors:** Sven C. J. van Dun, Mariëlle Verheul, Bart G. C. W. Pijls, Joffrey van Prehn, Henk Scheper, Federica Galli, Peter H. Nibbering, Mark G. J. de Boer

**Affiliations:** ^1^Department of Infectious Diseases, Leiden University Medical Center, Leiden, Netherlands; ^2^Department of Orthopedics, Leiden University Medical Center, Leiden, Netherlands; ^3^Department of Medical Microbiology, Leiden University Medical Center, Leiden, Netherlands; ^4^Leiden Institute of Physics, Leiden, Netherlands

**Keywords:** PJI, *Staphylococcus aureus*, MRSA, biofilm, atomic force microscopy (AFM)

## Abstract

**Introduction:**

One of the main causes of treatment failure in bacterial prosthetic joint infections (PJI) is biofilm formation. The topography of the biofilm may be associated with susceptibility to antimicrobial treatment. The aims of this study were to assess differences in topography of biofilms on different implant materials and the correlation thereof with susceptibility to antimicrobial treatment.

**Methods:**

Methicillin-resistant *Staphylococcus aureus* (MRSA) 7-day mature biofilms were generated on disks made from titanium alloys (Ti-6Al-7Nb and Ti-6Al-4V), synthetic polymer and orthopedic bone cement, commonly used in implant surgery. The surface topography of these implant materials and the biofilms cultured on them was assessed using atomic force microscopy. This provided detailed images, as well as average roughness (Ra) and peak-to-valley roughness (Rt) values in nanometers, of the biofilm and the material surfaces. Bacterial counts within biofilms were assessed microbiologically. Antimicrobial treatment of biofilms was performed by 24-h exposure to the combination of rifampicin and ciprofloxacin in concentrations of 1-, 5- and 10-times the minimal bactericidal concentration (MBC). Finally, treatment-induced differences in bacterial loads and their correlation with biofilm surface parameters were assessed.

**Results:**

The biofilm surfaces on titanium alloys Ti-6Al-7Nb (Ra = 186 nm) and Ti-6Al-4V (Ra = 270 nm) were less rough than those of biofilms on silicone (Ra = 636 nm). The highest roughness was observed for biofilms on orthopedic bone cement with an Ra of 1,551 nm. Interestingly, the roughness parameters of the titanium alloys themselves were lower than the value for silicone, whereas the surface of the bone cement was the roughest. Treatment with 1- and 5-times the MBC of antibiotics resulted in inter-material differences in colony forming units (CFU) counts, ultimately showing comparable reductions of 2.4–3.0 log CFU/mL at the highest tested concentration. No significant differences in bacterial loads within MRSA biofilms were observed between the various implant materials, upon exposure to increasing concentrations of antibiotics.

**Discussion:**

The surface parameters of MRSA biofilms were determined by those of the implant materials on which they were formed. The antibiotic susceptibility of MRSA biofilms on the various tested implant materials did not differ, indicating that the efficacy of antibiotics was not affected by the roughness of the biofilm.

## 1. Introduction

Orthopedic implants and other indwelling devices have improved quality of life for many patients. However, these implants can be accompanied by health risks, such as infection. Prosthetic joint infection (PJI) is the complication with the highest incidence rate for orthopedic implants, occurring in 0.5–2% of patients (Abad and Haleem, 2018; Beam and Osmon, 2018; Ahmed and Haddad, 2019). An infection at the implant site may originate from microbial contamination during surgery or from hematogenous seeding. PJI may present itself as either an acute or chronic infection (Dibartola et al., 2017; Zimmerli and Sendi, 2017; Beam and Osmon, 2018). A wide range of bacterial species can cause PJIs. *Staphylococcus aureus* is the microorganism most commonly associated with these infections. The site and material of an implant may influence the type of causative microorganisms and biofilm formation (Arciola et al., 2018). Treatment of patients with an acute PJI consists of surgical debridement of the implant and the infected tissue around it, followed by 6–12 weeks of antibiotic therapy. Nevertheless, failure rates for this treatment strategy are high (10–45%) (Scheper et al., 2021a). Treatment failure is often caused by surviving bacteria within a biofilm, formed on the implant (Gbejuade et al., 2015). The formation process starts by bacterial adhesion to the implant surface. This attachment varies based on the properties of the material and the expression of bacterial adhesins (Gristina and Costerton, 1985; Gristina et al., 1988; Ribeiro et al., 2012). Following adhesion, bacteria can cluster together via these adhesins and other cell wall proteins, forming microcolonies in the early biofilm stage. During the maturation process, bacterial polymers form an extracellular matrix (ECM) together with extracellular DNA of dead bacterial or host cells. This matrix builds up and allows for large structure formation. The ECM serves as a protective barrier against harmful environmental factors, such as antibiotics and host immune cells. Ultimately, bacteria may detach from the mature biofilm and disseminate throughout its surroundings (Zimmerli and Sendi, 2017; Arciola et al., 2018; Vestby et al., 2020). The scientific understanding of individual cells in biofilms and the biofilm system as a whole has developed over the years with the introduction of new analytical techniques. One of these techniques is atomic force microscopy (AFM), which has been used in industrial settings for several decades (Beech et al., 2002; Chatterjee et al., 2014; Grzeszczuk et al., 2020). However, in research on infectious diseases, specifically biofilm infections, AFM is yet to be explored. The AFM system senses differences in surface tension by interaction with the sample and changes in cantilever position and uses this to map surface topography and interaction force between tip and sample. Biofilm formation is not only dependent on the bacterial species, but on the solid substrate to which it is attached as well (Rodriguez et al., 2008; Oh et al., 2009). Various materials can be used to study these differences on a cellular level or in a mature biofilm system. In this way, insight can be gained into biofilm forming properties of clinically relevant materials which are used for prosthetics, catheters or other medical implants. The aims of this study were to assess differences in topography of biofilms on different, clinically relevant implant materials and the correlation thereof with susceptibility to antimicrobial treatment.

## 2. Materials and methods

### 2.1. Implant materials

Two titanium alloys, namely medical grade 5 titanium-7% aluminum-6% niobium (TAN; ISO 5832/11) disks (diameter 5 mm, height 1.5 mm) and medical grade 5 titanium-6% aluminum-4% vanadium (TAV; ISO 5832/3, Braun) disks (diameter 4 mm, height 1.5 mm), were used. Orthopedic bone cement (Palacos^®^ fast R + G, 66057601, Heraeus, Hanau, Germany) disks (diameter 4 mm, height 2 mm) containing 0.62 g gentamicin per batch were produced by spreading the mixture over mold strips with the correct size. After drying and hardening, disks were removed from the strips. We used antibiotic-impregnated bone cement, as opposed to pristine bone cement, since impregnated cement is very commonly used in patients. Non-impregnated, pristine bone cement is less common. Furthermore, silicone elastomer disks (punched with biopsy punch Ø 4 mm, Stiefel, #2957 out of a silicone elastomer sheet, SI303060, sheet 600 mm×600 mm, Goodfellow Cambridge Ltd., Huntingdon, UK #572-667-36) were used to assess synthetic polymers. All materials were selected based on their relevance in the context of clinical implant-associated infections and are shown in Supplementary Figure 2. All disks were made specifically to fit in 96-well plates.

### 2.2. Bacterial culture

Methicillin-resistant *S. aureus* (MRSA) strain LUH14616 (NCCB100829) stocks were stored in 20% glycerol at −80°C until use. Prior to experiments, stocks were thawed and spread on trypticase soy agar with 5% sheep blood plates (Biomerieux, Marcy-l’Étoile, France, 43009) and cultured aerobically overnight at 37°C. Thereafter, bacteria were cultured aerobically at 37°C to mid-log phase in tryptic soy broth (Oxoid, Basingstoke, Hampshire, UK, CM0129) for 2.5 h under continuous spin at 200 rotations per minute. Bacteria were concentrated by centrifugation at 1,000×*g* for 10 min and washed with phosphate-buffered saline (PBS, pH 7.4). Ultimately, the bacteria were resuspended in brain heart infusion (BHI) broth (Oxoid, Basingstoke, Hampshire, UK, CM1135B) to the required concentrations based on the optical density at 600 nm, measured using Ultrospec 10 (GE Healthcare Bio-Sciences AB, Cambridge, UK).

### 2.3. Antibacterial agents

Rifampicin (R3501, Sigma-Aldrich, 822.94 g/mol) and ciprofloxacin (PHR 1044, Sigma-Aldrich, 385.82 g/mol) were stored at −20°C until use. Stock solutions of 4.0 g/L and 25.6 g/L were prepared, respectively. Concentrations corresponding to 10-times the minimal bactericidal concentration (MBC) for the MRSA strain (1,280 g/L ciprofloxacin, 10 mg/L rifampicin) were used, as well as 5- and 1-time the MBC. Here, the MBC was defined as the lowest concentration needed to kill 99.9% of planktonic bacteria compared to untreated controls.

### 2.4. Anti-biofilm assay and CFU counts

A validated model (Scheper et al., 2021b) was used, in which implant material disks were placed in wells of a 96-well plate. Bacteria were diluted in BHI to obtain a suspension of mid log-phase bacteria (1×10^7^ CFU/mL). A volume of 100 μL of this suspension was added to each well of a polystyrene flat bottom microplate (Greiner Bio-One, Frickenhausen, Germany) with implant material disks. Plates were incubated aerobically for 24 h or 7 days at 37°C to form immature or mature biofilms, respectively. Next, 96-well plates with immature biofilms were sealed with non-breathable plastic film sealers (Amplistar, Westburg, Germany). Breathable rayon film sealers (VWR European, Leuven, Belgium) were used for mature biofilms. After incubation, bacterial suspensions were discarded from the wells. Subsequently, the wells were gently washed twice with 100 μL of PBS to remove the non-adherent bacterial cells. Disks with biofilm were transferred to new polystyrene flat bottom 96-well plates, to lose any leftover non-adherent bacteria in the old 96-well plates. The anti-biofilm assays were performed on mature biofilms, as these are most relevant to biofilm-associated infections. The remaining biofilms were subsequently exposed to 1-, 5- or 10-times the MBC of the antibiotics rifampicin (RIF) and ciprofloxacin (CIP) (Le Vavasseur and Zeller, 2022) for 24 h at 37°C. Untreated biofilm controls were included to assess treatment efficacy. Medium and diluent controls without bacteria were used to monitor possible contamination. After treatment, culture medium was removed, and wells were gently washed twice with 100 μL of PBS. Disks were transferred to new flat bottom polystyrene 96-well plates containing 100 μL of PBS. Plates were sealed with aluminum stickers and sonicated for 10 min at 40 kHz in Bransonic^®^ M Mechanical Bath 1800 (Branson Ultrasonics, Brookfield, CT, USA) to detach biofilms from the implant material. Thereafter, well contents were resuspended to acquire all leftover biofilm material. Bacterial suspensions were transferred to round-bottom polystyrene 96-well plates (Greiner Bio-One, Frickenhausen, Germany) and 10-times dilution series were made. Bacterial suspensions of all dilutions were plated on Muller-Hinton agar plates (Oxoid, Basingstoke, Hampshire, UK, CM0337) in duplicate. After the spots were dried, plates were incubated aerobically overnight at 37°C. Thereafter, colony growth was assessed and colony forming units (CFU) were counted. This experiment was repeated multiple times with 2 or 3 disks per material per variation, for a total of *N* = 17 disks per material and per variation.

### 2.5. Imaging by atomic force microscopy

Atomic force microscopy (AFM) images of implant materials were acquired using a JPK NanoWizard IV (Berlin, Germany) integrated with an up-right microscope (JPK “TopViewOptics” with Navitar long working distance zoom lens). Measurements were performed in ambient conditions and in intermittent contact mode, or AC mode, using uncoated silicon ACL cantilevers from AppNano (Mountain View, CA, USA) with typical resonance frequencies of 160–225 kHz, a spring constant of 36—90 N/m and average nominal tip radius of 6 nm. Scan speeds ranged between 0.2 and 0.4 Hz and total scan areas of 100 μm×100 μm were imaged per measurement to obtain surface parameters. Scans of 5 μm×5 μm were taken to obtain more detailed images of implant material and biofilm surfaces. Images were made at 5 random locations per disk for a total of *N* = 15 images per material and per timepoint. Surface roughness (Ra) in nm, as well as peak-to-valley roughness (Rt) or total height difference in nm were determined (Box 1). The captured images were processed using JPKSPM Data Processing software version 6.1.191 (JPK BioAFM, Bruker Nano GmbH, Berlin, Germany).

**BOX 1 Surface roughness and topography.**

Surface roughness can be defined as the measure of total irregularities on a surface. It plays an important role in the interaction between a surface and its environment. It can be calculated by measuring the relative smoothness of a surface’s profile, otherwise known as the arithmetical mean roughness (Ra). It can be calculated using a standard formula: $R\,a=\frac{1}{L}\,\int_{0}^{L}\left\{f\,\left(x\right)\right\}\,dx$ where *L* stands for the length of the evaluated surface and *f(x)* expresses the roughness curve. From this formula, the root mean square roughness (RMS) can be calculated: $R\,M\,S=\sqrt{\left[\frac{1}{L}\,\int_{0}^{L}\left\{f\,{\left(x\right)}^{2}\right\}\,dx\right]}$. Both formulas can be representations of the roughness of a surface. However, a single outlier will affect the RMS value more than the Ra value. The average maximum height of a surface is known as Rz and represents the sum of the maximum peak height Zp and maximum valley depth Zv of a surface profile within the measured sampling length: *Rz* = *Zp* + *Zv*. The total height or peak-to-valley roughness of a surface can be characterized by: *Rt* = max(*Zp*) + max(*Zv*). This is the vertical distance between the highest and lowest points of a surface profile. The difference between Rz and Rt can be explained by the fact that Rt is conducted against the full evaluation length, whereas Rz is measured on a specific length. Therefore the basic rule is Rt ≥ Rz. In this study, we made use of the parameters Ra and Rt to characterize material and biofilm surfaces.

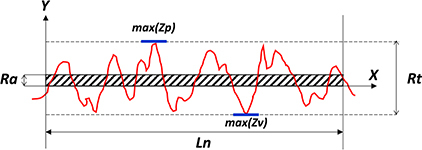

Surface profile with mean roughness Ra and total height Rt over the evaluation length Ln. max (Zp) indicates the highest peak of the profile, and max (Zv) indicates the lowest valley of the profile.

### 2.6. Biofilm fixation prior to AFM

Immature and mature staphylococcal biofilms were fixed with 0.1% (v/v; in MilliQ) glutaraldehyde for 4 h at room temperature and samples were left to dry overnight after removing fixative. Additionally, mature biofilms treated with antibiotics, as described in section “2.4. Anti-biofilm assay and CFU counts,” were also fixed. After fixation, biofilms were imaged in the same manner as previously described. Biofilm Ra and Rt were assessed with JPKSPM Data Processing software to quantify the topographical changes observed.

### 2.7. Statistical analysis

Differences in surface Ra and Rt between materials and biofilms were determined with their corresponding 95% confidence intervals. Data with a non-normal distribution, such as CFU counts, were log10 transformed to obtain a normal distribution (O’Toole, 2011). Mann–Whitney and Kruskall–Wallis statistical tests were performed to assess statistical differences between findings. In line with recent recommendations, means, mean differences and corresponding confidence intervals (CIs) were reported, while *p*-values are not reported (Amrhein et al., 2019). All calculations and statistical tests were performed using GraphPad Prism version 9.3.1 (GraphPad Software, San Diego, CA, USA).

## 3. Results

### 3.1. Surface topography and roughness parameters of different implant materials

**FIGURE 1:**
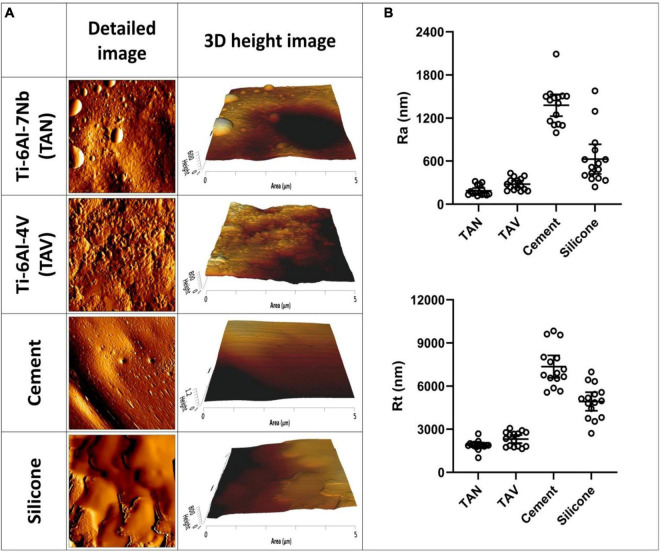
Atomic force microscopy images of various implant materials. **(A)** Per material and per timepoint a detailed 5 μm×5 μm image and a 3D height image is given. **(B)** The corresponding surface parameters of these materials are shown. On the *y*-axis, the surface roughness Ra and peak-to-valley roughness Rt are shown. On the *x*-axis, the following materials are shown: Ti-6Al-7Nb (TAN); Ti-6Al-4V (TAV); orthopedic bone cement and silicone. Each datapoint represents one random location on a disk (3 disks; 5 areas each; *N* = 15). Horizontal lines indicate the means and error bars indicate 95% confidence interval.

Atomic force microscopy imaging of a standard test set of implant materials was performed to characterize surface topography. The differences between materials were optimally visualized with detailed 5 μm×5 μm AFM images (Figure 1A), showing variability in surface characteristics. For example, the porous nature of the orthopedic bone cement resulted in small holes and grooves on the surface, and the soft nature of the silicone resulted in less defined features. Corresponding surface parameters showed significant differences between materials (Figure 1B). The mean Ra value of the titanium alloy TAN was 187 nm and mean Rt was 1,883 nm. For TAV, Ra was 279 nm and Rt was 2,313 nm. Measurements showed overlap for both parameters, with no significant differences between the two materials. Both titanium alloys were less rough than silicone, with Ra and Rt values of 627 nm and 4,929 nm, respectively. The highest roughness was observed for orthopedic bone cement, with 1,379 nm for Ra and 7,354 nm for Rt. Additionally, the variation in Ra and Rt values is larger for orthopedic bone cement and silicone compared to both the titanium alloys, as indicated by the larger error bars (Supplementary Table 1).

### 3.2. Surface topography and roughness related to biofilm maturity

**FIGURE 2:**
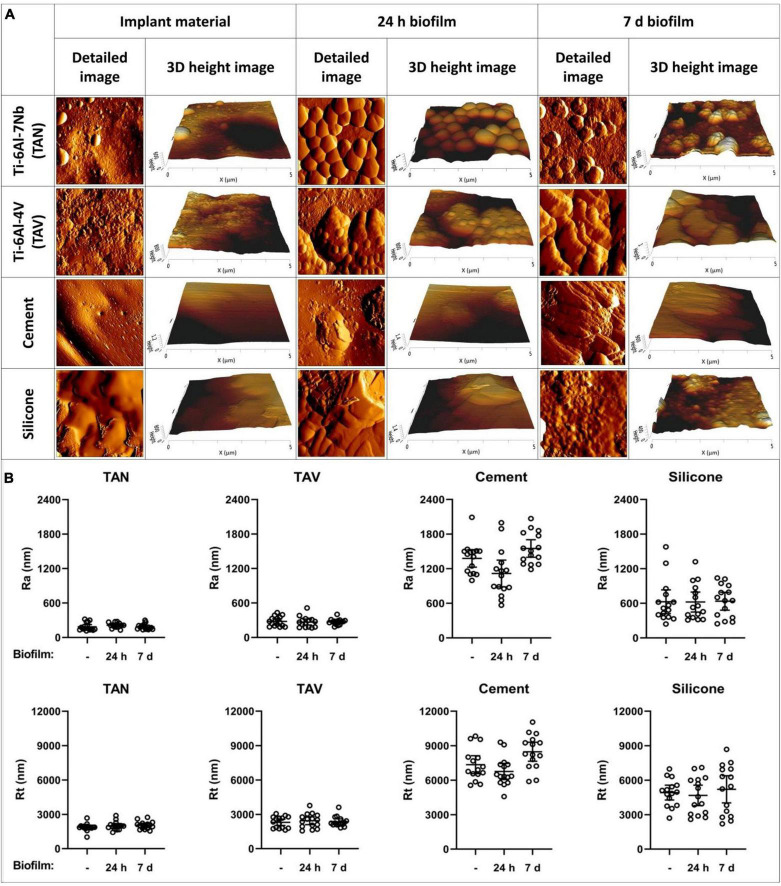
Atomic force microscopy images of various implant materials. **(A)** At different timepoints: at baseline without bacteria, with 24-h biofilm and with 7-day biofilm. Per material and per timepoint a detailed 5 μm×5 μm image and a 3D height image are shown. **(B)** Surface parameters of the implant materials Ti-6Al-7Nb (TAN) Ti-6Al-4V (TAV), orthopedic bone cement and silicone. On the *y*-axis, the surface roughness Ra and peak-to-valley roughness Rt are shown. On the *x*-axis, the following biofilm timepoints are shown:–(no biofilm); 24 h biofilm; 7 days biofilm. Each datapoint represents one disk (3 disks; 5 areas each; *N* = 15). Horizontal lines indicate the means and error bars indicate 95% confidence intervals.

To investigate structural differences in the biofilm maturation process, we fixed 24 h and 7-day biofilms on the beforementioned materials. Examples of the various timepoints are shown in Figure 2A. Structural differences between the immature and mature biofilms were observed, with increasing complexity in biofilm characteristics. To clarify, staphylococcal biofilms cultured on TAN disks for 24 h showed attachment and clumping of individual bacterial cells on the surface, suggesting an early phase of the biofilm formation. The mature, 7-day biofilm showed large, multilayered structures with ECM covering the individual bacterial cells, as can be seen for TAV.

### 3.3. Influence of implant materials on staphylococcal biofilm surface parameters

Next, we investigated whether the surface parameters of the implant materials influenced those of the biofilms cultured on these materials for 24 h or 7 days. TAN disks showed the lowest mean Ra and Rt values in all timepoints, not reaching above 212 nm and 2,054 nm, respectively (Figure 2B). The measurements showed no significant differences to those of TAV, clustering the titanium alloys together. The non-metal implant materials showed the highest mean values in all measurements (Supplementary Table 1). This was expected, since the titanium alloys are comparable to the relatively smooth topography of their clinically used forms. The surface Ra and Rt of immature and mature biofilms were similar to the parameters of the materials without biofilm (Figure 2B). Therefore, the surface parameters Ra and Rt for immature and mature biofilms did not differ statistically on any of the four tested implant materials. This suggests that the staphylococcal biofilm surface parameters are dictated by the corresponding implant surface topography.

### 3.4. The effect of antibiotic treatment on MRSA in mature biofilms on different implant materials

**FIGURE 3:**
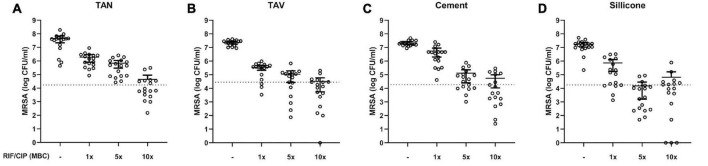
Bacterial load (log CFU/mL) of mature 7-day MRSA biofilms adherent to various implant material disks: **(A)** titanium-aluminum-niobium (TAN); **(B)** titanium-aluminum-vanadium (TAV); **(C)** orthopedic bone cement and **(D)** silicone after 24 h exposure to 1-, 5- and 10-times the MBC of rifampicin (RIF) and ciprofloxacin (CIP). Results are shown as individual values (*N* = 17), horizontal lines indicated means and error bars indicate 95% CI. The dotted line indicates 99.9% eradication threshold. CFU: colony forming units; MBC: minimal bactericidal concentration.

As AFM showed structural differences in staphylococcal biofilms, the effect of 24-h exposure to rifampicin and ciprofloxacin in various concentrations was assessed. This was done to gain insight in the potential differences in susceptibility to treatment of mature biofilms (methods section “2.4. Anti-biofilm assay and CFU counts”). For this, biofilms were cultured onto the standard test set of implant materials. Exposure to increasing concentrations of the antibiotics resulted in a dose-dependent decrease of bacterial loads for all materials compared to untreated controls (Figure 3). All materials showed comparable bacterial loads with limited variation compared to their mean in the absence of antibiotic treatment, while inter-material differences in susceptibility to the lower concentrations of antibiotics (1 and 5× MBC) were observed (Supplementary Figure 1). Results reveal comparable CFU counts on the implant materials at the highest tested concentration (10× MBC) of antibiotics. The remaining mean bacterial loads all ranged from 6.58×10^4^ CFU/mL to 2.25×10^4^ CFU/mL.

**FIGURE 4:**
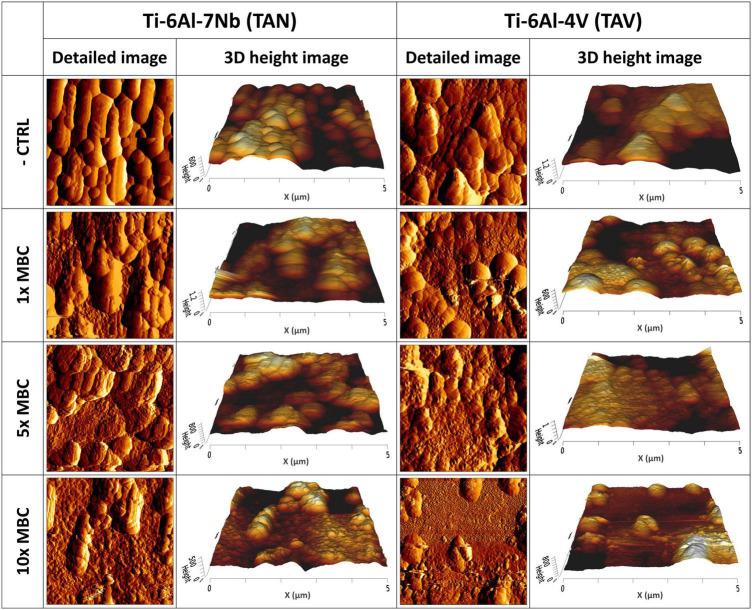
Atomic force microscopy images of mature 7-day biofilms cultured on implant materials: **(Left)** Ti-6Al-7Nb (TAN); **(Right)** Ti-6Al-4V (TAV) after 24 h exposure to 1-, 5- and 10-times the MBC of rifampicin (RIF) and ciprofloxacin (CIP). Per material and per concentration a detailed 5 μm×5 μm image and a 3D height image are shown.

In addition, we assessed the effect of the antibiotics on the topology of biofilms on titanium alloys (TAN and TAV) by using AFM (Figure 4). The controls showed 7-day biofilms with multiple layers and presence of ECM. Biofilms exposed to 1× MBC of antibiotics were characterized by the presence of distinguishable layers and individual bacterial cells, with more uneven surfaces compared to controls. Exposure to 5× MBC resulted in less pronounced structure of the biofilm with an even rougher surface compared to controls and 1× MBC exposure. Individual bacterial cells were more difficult to distinguish from the surroundings. Exposure of biofilms to 10× MBC led to a major reduction in bacterial cell numbers per image, compared to controls. This reduction correlates with the reduction in CFU counts shown in Figure 3. Virtually all remaining bacterial cells displayed a heavily disrupted surface.

## 4. Discussion

The burden of biofilm-associated infections, including PJIs, on healthcare and society as a whole emphasizes the need for characterization of biofilms. This information is instrumental in identifying treatment targets and developing novel anti-biofilm agents. Here, we characterized the surface topography of various implant materials and staphylococcal biofilms formed on these materials *in vitro* using AFM. Additionally, we compared the biofilm formation on these implant materials as well as the susceptibility of the bacteria within biofilms to antibiotics using microbiological assays.

The implant materials used in this study are a selection of an expanding list of materials used in joint prostheses and indwelling devices. Existing studies have already compared material properties of TAN and TAV based on roughness, durability and toxicity (Challa et al., 2013; Fellah et al., 2014; Mamoun et al., 2016). We selected materials commonly used in the clinic to gain insight in the differences between metal and non-metal implant material. To the best of our knowledge, biofilm formation on orthopedic bone cement in this format has not been previously studied, while knowledge on this commonly used bone-implant interface material is needed. The use of antibiotic-impregnated bone cement, as opposed to pristine bone cement was mentioned in methods section “2.1. Implant materials.” Additionally, the MRSA strain that was used in this study (LUH14616) has been tested resistant to gentamicin. Therefore, it would not have differed for the growth of this strain and development of a biofilm on the cement disks, with or without the gentamicin impregnation. Research on orthopedic bone cement should be aimed at gaining more insight into its role in PJIs as several studies reported higher revision rates in cemented implants compared to cementless implants (Engesaeter et al., 2006; Quispel et al., 2021). Several other implant materials were not included due to lack of availability or difficulties in processing, such as cobalt-chromium alloys or ceramics. The comparison of disks from the same biomaterial, but with modified surface roughness parameters, could be of interest to further study the direct effect on the topology of the biofilm.

Exposure of mature staphylococcal biofilms to rifampicin and ciprofloxacin in various high concentrations resulted in a dose-dependent reduction in bacterial loads, with statistical differences between treated and untreated biofilms. Treatment with the highest tested concentration (10× MBC) even resulted in a decrease of CFU counts below the 99.9% eradication threshold for multiple disks per material. Despite this decrease, these findings cannot be interpreted as clinically relevant, since relatively high bacterial loads remain after antibiotic treatment. The surviving bacterial fractions, with mean values of around 4-log CFU/mL, may expand rapidly, considering the growth rate of *S. aureus* (Dalhoff, 1985). This may result in recurrent and chronic infections. Moreover, these fractions have been exposed to high concentrations of antibiotics, potentially limiting treatment options due to resistance and/or tolerance development. This emphasizes the need for alternative treatment strategies against PJIs and other biofilm-associated infections.

The presence of a tolerant or resistant fraction of bacteria may be related to the presence of small-colony variants (SCVs) of *S. aureus*. This phenomenon has been described as a common mechanism to increase survival chances, where the bacteria change the electrochemical gradient of the membrane, making them more resilient in stressful environments, e.g., under antibiotic pressure (Melter and Radojeviè, 2010). In this study, these SCVs were frequently observed after treatment with high concentrations of antibiotics or after culture on orthopedic bone cement, which has antibiotic mixed into it. The SCV phenotype and metabolism remains incompletely understood, and its occurrence in our study needs to be explored further.

Imaging with AFM revealed differences in surface roughness and height between the tested implant materials. These differences translated further onto the surface topography of the biofilms cultured on the implant materials. Our findings suggest that biofilm characteristics are dictated by the material on which they are formed. For example, when this surface has a high roughness, the bacterial cells will follow this topography in the early phases of biofilm development. When the biofilm matures over time, this clustering of cells and production of ECM influences the formation of the biofilm and therefore changes its surface topography. The role of ECM in biofilm structuring and physiology has been studied before (Dragoš and Kovács, 2017; Bowen et al., 2018; Karygianni et al., 2020), but several aspects remain poorly understood and will require more research.

A previous study (Jordana et al., 2018) classified implant materials based on their roughness. Since similar materials are used in these implants, this could be compared to our materials. Our TAN and TAV disks could be considered smooth (Ra < 0.5 μm), silicone could be considered minimally rough (Ra between 0.5 and 1.0 μm) and orthopedic bone cement could be considered moderately rough (Ra between 1.0 and 2.0 μm). They observed that increased roughness on dental implants resulted in an increased peri-implantitis rate. This contrasts with our findings, since we did not observe differences between bacterial loads on the various implant materials. However, the presence of bacteria, and thus chance of infection, in the oral cavity cannot be translated to that of the joints.

The development of staphylococcal biofilms on the implant disks resulted in structural differences based on the incubation period. Biofilms cultured for 24 h showed clustering of bacterial cells under AFM, suggesting a phase where microcolonies are formed to make an immature biofilm. Biofilms cultured for 7 days appeared as large, matrix-encased bacterial clusters which indicates a mature biofilm. Existing studies on staphylococcal biofilms have classified culture for as short as 24 h as mature biofilms (Höing et al., 2018; Cascioferro et al., 2019), with studies even mentioning 4 h culture as an immature biofilm (O’Toole, 2011). To further support our approach, a biofilm development timeline experiment could be performed, in which implant material disks with biofilms are fixed every 24 h, starting as early as 1 h after start of culture up to 7 days or longer. This could show an increase in complexity and structure formation over time. Based on previous experiments in our lab (data not shown), the bacterial load within biofilms should remain similar throughout the incubation period. This indicates that the biofilm formation is focused on production of ECM by the present bacteria, which helps these bacterial cells to maintain their position and remain viable.

This study has some limitations that need to be considered. Atomic force microscopy has proven to be an excellent tool in characterizing implant material and biofilm surface topography, providing detailed images as well as information on surface parameters (Beech et al., 2002). It allows for work under ambient conditions with minimal pretreatment, limiting the potential for artifacts. However, the three-dimensional scanning tip used in AFM provides a 3D image output, but only of the fixed outer surface. Therefore, AFM cannot visualize inner biofilm structures and composition, making it insufficient to completely characterize biofilms by itself. This could be circumvented by combining AFM with another imaging technique, such as confocal laser scanning microscopy (CLSM). Second, biofilm fixation by glutaraldehyde may be accompanied by changes in cell surface conformation and loss of viability (Liu et al., 2012). To limit this potential adverse effect and artifact formation before or during imaging, we used a very low concentration of 0.1% glutaraldehyde. Third, the antibiotic concentrations that have been used in this study are based on minimum inhibitory and bactericidal concentrations for planktonic bacteria. Determining the exact concentrations needed to eradicate biofilms can be challenging, and may vary between antibiotics (Macia et al., 2014). This can even differ based on the maturity of the biofilm (Amorena et al., 1999). However, our approach of 7 day generated biofilms is infrequently described in literature. As observed in the AFM images, the mature biofilms showed increased complexity and presence of ECM, which could limit the antibiotic penetration and efficacy. Ultimately, we used these antibiotic concentrations to be able to compare our *in vitro* assay to concentrations that are used in the clinical setting. Fourth, we studied only one bacterial strain and a combination of two antibiotics in this *in vitro* study. To further investigate the role of implant materials in biofilm formation and treatment susceptibility, more strains need to be tested with a range of antibiotic resistance profiles and biofilm-forming capabilities. Additionally, more antibiotics, antimicrobials and other treatment strategies need to be assessed to further investigate how surface topography and biofilm structure may affect its susceptibility to said treatments. Another interesting aspect that requires further research, is the relation between the biofilm surface roughness and topology with bacterial activity. This could be studied by combining AFM with other techniques that give insight in the bacterial activity within the biofilm (Holman et al., 2009; Yeor-Davidi et al., 2020).

To summarize, our standard test set of implant materials was characterized based on the surface parameters Ra and Rt using AFM. Surface topography and roughness parameters differed between implant materials. These differences in surface parameters between implant materials dictate biofilm roughness and characteristics generated on the tested materials. Despite structural and statistical differences in biofilm surface topography, the susceptibility of the MRSA strain to rifampicin and ciprofloxacin in mature biofilms was similar. These results indicated that there is no direct association between antibiotic susceptibility of mature MRSA biofilms and the surface topography of the materials on which they were cultured.

## Author’s Note

Preliminary data were submitted in abstract form to the 33rd ECCMID congress, Copenhagen, to be held from 15–18 April 2023.

## Data availability statement

The raw data supporting the conclusions of this article will be made available by the authors, without undue reservation.

## Author contributions

MB, PN, and BP contributed to conception and design of the study. SD and MV conducted the experiments. JP and HS supported data retrieval and experimental development. FG supervised the conducted experiments using atomic force microscopy. SD, MB, PN, BP, and MV performed the data analysis. SD wrote the first draft of the manuscript. All authors contributed to manuscript revision, read, and approved the submitted version.

## References

[B1] Abad C. L. Haleem A. (2018). Prosthetic joint infections: An update. *Curr. Infect. Dis. Rep.* 20:15. 10.1007/s11908-018-0622-0 29789958

[B2] Ahmed S. S. Haddad F. S. (2019). Prosthetic joint infection. *Bone Joint Res.* 8 570–572. 10.1302/2046-3758.812.BJR-2019-0340 31832177 PMC6888735

[B3] Amorena B. Gracia E. Monzón M. Leiva J. Oteiza C. Pérez M. (1999). Antibiotic susceptibility assay for *Staphylococcus aureus* in biofilms developed *in vitro*. *J. Antimicrob. Chemother.* 44 43–55. 10.1093/jac/44.1.43 10459809

[B4] Amrhein V. Greenland S. McShane B. (2019). Scientists rise up against statistical significance. *Nature* 567 305–307. 10.1038/d41586-019-00857-9 30894741

[B5] Arciola C. R. Campoccia D. Montanaro L. (2018). Implant infections: Adhesion, biofilm formation and immune evasion. *Nat. Rev. Microbiol.* 16 397–409. 10.1038/s41579-018-0019-y 29720707

[B6] Beam E. Osmon D. (2018). Prosthetic joint infection update. *Infect. Dis. Clin. North Am.* 32 843–859. 10.1016/j.idc.2018.06.005 30241717

[B7] Beech I. B. Smith J. R. Steele A. A. Penegar I. Campbell S. A. (2002). The use of atomic force microscopy for studying interactions of bacterial biofilms with surfaces. *Colloids Surf B Biointerfaces* 23 231–247. 10.1016/S0927-7765(01)00233-8

[B8] Bowen W. H. Burne R. A. Wu H. Koo H. (2018). Oral biofilms: Pathogens, matrix, and polymicrobial interactions in microenvironments. *Trends Microbiol.* 26 229–242. 10.1016/j.tim.2017.09.008 29097091 PMC5834367

[B9] Cascioferro S. Parrino B. Petri G. L. Cusimano M. G. Schillaci D. Di Sarno V. (2019). 2,6-Disubstituted imidazo[2,1-b][1,3,4]thiadiazole derivatives as potent staphylococcal biofilm inhibitors. *Eur. J. Med. Chem.* 167 200–210. 10.1016/j.ejmech.2019.02.007 30772604

[B10] Challa V. S. Mali S. Misra R. D. (2013). Reduced toxicity and superior cellular response of preosteoblasts to Ti-6Al-7Nb alloy and comparison with Ti-6Al-4V. *J. Biomed. Mater. Res. A* 101 2083–2089. 23349101 10.1002/jbm.a.34492

[B11] Chatterjee S. Biswas N. Datta A. Dey R. Maiti P. (2014). Atomic force microscopy in biofilm study. *Microscopy* 63 269–278. 10.1093/jmicro/dfu013 24793174

[B12] Dalhoff A. (1985). Differences between bacteria grown in vitro and in vivo. *J. Antimicrob. Chemother.* 15 175–195. 10.1093/jac/15.suppl_A.175 2858465

[B13] Dibartola A. C. Swearingen M. C. Granger J. F. Stoodley P. Dusane D. H. (2017). Biofilms in orthopedic infections: A review of laboratory methods. *APMIS* 125 418–428. 10.1111/apm.12671 28407424

[B14] Dragoš A. Kovács ÁT. (2017). The peculiar functions of the bacterial extracellular matrix. *Trends Microbiol.* 25 257–266. 10.1016/j.tim.2016.12.010 28089324

[B15] Engesaeter L. B. Espehaug B. Lie S. A. Furnes O. Havelin L. I. (2006). Does cement increase the risk of infection in primary total hip arthroplasty? Revision rates in 56,275 cemented and uncemented primary THAs followed for 0-16 years in the Norwegian arthroplasty register. *Acta Orthop.* 77 351–358. 10.1080/17453670610046253 16819671

[B16] Fellah M. labaïZ M. Assala O. Dekhil L. Zerniz N. Alain I. (2014). Tribological behavior of biomaterial for total hip prosthesis. *Mater. Tech.* 102 6–7. 10.1051/mattech/2014027

[B17] Gbejuade H. O. Lovering A. M. Webb J. C. (2015). The role of microbial biofilms in prosthetic joint infections. *Acta Orthop.* 86 147–158. 10.3109/17453674.2014.966290 25238433 PMC4404764

[B18] Gristina A. G. Costerton J. W. (1985). Bacterial adherence to biomaterials and tissue. The significance of its role in clinical sepsis. *J. Bone Joint Surg. Am.* 67 264–273. 10.2106/00004623-198567020-00014 3881449

[B19] Gristina A. G. Naylor P. Myrvik Q. (1988). Infections from biomaterials and implants: A race for the surface. *Med. Prog. Technol.* 14 205–224. 2978593

[B20] Grzeszczuk Z. Rosillo A. Owens Ó Bhattacharjee S. (2020). Atomic force microscopy (AFM) as a surface mapping tool in microorganisms resistant toward antimicrobials: A mini-review. *Front. Pharmacol.* 11:517165. 10.3389/fphar.2020.517165 33123004 PMC7567160

[B21] Höing B. Kirchhoff L. Arnolds J. Hussain T. Buer J. Lang S. (2018). Bioactive glass granules inhibit mature bacterial biofilms on the surfaces of cochlear implants. *Otol. Neurotol.* 39 e985–e991. 10.1097/MAO.0000000000002021 30334871

[B22] Holman H. Y. Miles R. Hao Z. Wozei E. Anderson L. M. Yang H. (2009). Real-time chemical imaging of bacterial activity in biofilms using open-channel microfluidics and synchrotron FTIR spectromicroscopy. *Anal. Chem.* 81 8564–8570. 10.1021/ac9015424 19775125

[B23] Jordana F. Susbielles L. Colat-Parros J. (2018). Periimplantitis and implant body roughness: A systematic review of literature. *Implant Dent.* 27, 672–681. 30475272 10.1097/ID.0000000000000834

[B24] Karygianni L. Ren Z. Koo H. Thurnheer T. (2020). Biofilm matrixome: Extracellular components in structured microbial communities. *Trends Microbiol.* 28 668–681. 10.1016/j.tim.2020.03.016 32663461

[B25] Le Vavasseur B. Zeller V. (2022). Antibiotic therapy for prosthetic joint infections: An overview. *Antibiotics* 11:486. 10.3390/antibiotics11040486 35453237 PMC9025623

[B26] Liu B. Y. Zhang G. M. Li X. L. Chen H. (2012). Effect of glutaraldehyde fixation on bacterial cells observed by atomic force microscopy. *Scanning* 34 6–11. 10.1002/sca.20269 21898456

[B27] Macia M. D. Rojo-Molinero E. Oliver A. (2014). Antimicrobial susceptibility testing in biofilm-growing bacteria. *Clin. Microbiol. Infect.* 20 981–990. 10.1111/1469-0691.12651 24766583

[B28] Mamoun F. Omar A. Mohamed L. Leila D. Iost A. (2016). “Comparative study on tribological behavior of Ti-6Al-7Nb and SS AISI 316L alloys, for total hip prosthesis,” in *Proceedings of the TMS 2014: 143rd Annual Meeting & Exhibition*, (Cham: Springer International Publishing). 10.1007/978-3-319-48237-8_32

[B29] Melter O. Radojeviè B. (2010). Small colony variants of *Staphylococcus aureus*–review. *Folia Microbiol.* 55 548–558. 10.1007/s12223-010-0089-3 21253898

[B30] Oh Y. J. Lee N. R. Jo W. Jung W. K. Lim J. S. (2009). Effects of substrates on biofilm formation observed by atomic force microscopy. *Ultramicroscopy* 109 874–880. 10.1016/j.ultramic.2009.03.042 19394143

[B31] O’Toole G. A. (2011). Microtiter dish biofilm formation assay. *J. Vis. Exp.* 47:2437. 10.3791/2437-v PMC3182663 21307833

[B32] Quispel C. R. Duivenvoorden T. Beekhuizen S. R. Verburg H. Spekenbrink-Spooren A. Van Steenbergen L. N. (2021). Comparable mid-term revision rates of primary cemented and cementless total knee arthroplasties in 201,211 cases in the Dutch arthroplasty register (2007-2017). *Knee Surg. Sports Traumatol. Arthrosc.* 29 3400–3408. 10.1007/s00167-020-06183-2 32862239

[B33] Ribeiro M. Monteiro F. J. Ferraz M. P. (2012). Infection of orthopedic implants with emphasis on bacterial adhesion process and techniques used in studying bacterial-material interactions. *Biomatter* 2 176–194. 10.4161/biom.22905 23507884 PMC3568104

[B34] Rodriguez A. Autio W. R. McLandsborough L. A. (2008). Effects of contact time, pressure, percent relative humidity (%RH), and material type on listeria biofilm adhesive strength at a cellular level using atomic force microscopy (AFM). *Food Biophysics.* 3 305–311. 10.1007/s11483-008-9085-4

[B35] Scheper H. Gerritsen L. M. Pijls B. G. Van Asten S. A. Visser L. G. De Boer M. G. J. (2021a). Outcome of debridement, antibiotics, and implant retention for *Staphylococcal* hip and knee prosthetic joint infections, focused on rifampicin use: A systematic review and meta-analysis. *Open Forum Infect. Dis.* 8:ofab298. 10.1093/ofid/ofab298 34258321 PMC8271145

[B36] Scheper H. Wubbolts J. M. Verhagen J. A. M. de Visser A. W. van der Wal R. J. P. Visser L. G. (2021b). SAAP-148 eradicates MRSA persisters within mature biofilm models simulating prosthetic joint infection. *Front. Microbiol.* 12:625952. 10.3389/fmicb.2021.625952 33584628 PMC7879538

[B37] Vestby L. K. Grønseth T. Simm R. Nesse L. L. (2020). Bacterial biofilm and its role in the pathogenesis of disease. *Antibiotics* 9:59. 10.3390/antibiotics9020059 32028684 PMC7167820

[B38] Yeor-Davidi E. Zverzhinetsky M. Krivitsky V. Patolsky F. (2020). Real-time monitoring of bacterial biofilms metabolic activity by a redox-reactive nanosensors array. *J. Nanobiotechnol.* 18:81. 10.1186/s12951-020-00637-y 32448291 PMC7247256

[B39] Zimmerli W. Sendi P. (2017). Orthopaedic biofilm infections. *APMIS* 125 353–364. 10.1111/apm.12687 28407423

