# Supplementary material for: Influence of surface characteristics of implant materials on MRSA biofilm formation and effects of antimicrobial treatment
Source: Front Microbiol. 2023 Apr 20;14:1145210. doi: 10.3389/fmicb.2023.1145210 (PMC10159048; doi:10.3389/fmicb.2023.1145210)
Supplement: Supplementary file 1 [file Image_1.pdf]

Supplementary Material

Influence of surface characteristics of implant materials on MRSA biofilm formation and effects of antimicrobial treatment

Authors:
S.C.J. van Dun[1*], M. Verheul[1], B.G.C.W. Pijls[2], J. van Prehn[3], H. Scheper[1], F. Galli[4], P.H. Nibbering[1], M.G.J. de Boer[1]

*__Corresponding author:__
S.C.J. van Dun
Department of Infectious Diseases, Room E5-07
Address: Leiden University Medical Center, Albinusdreef 2, 2300 RC Leiden, the Netherlands
Tel: +31715261779
Email: s.c.j.van_dun@lumc.nl

1. Bactericidal activity of different antibiotic concentrations against mature biofilms

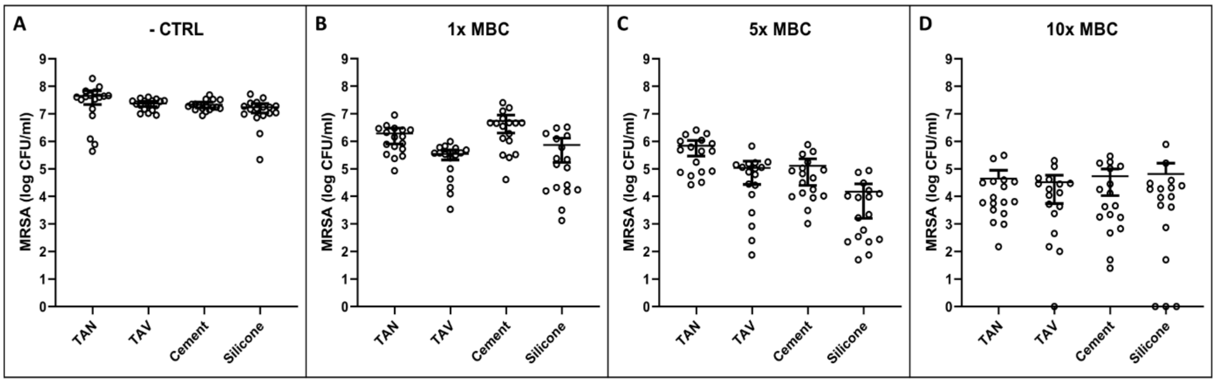

Supplementary Figure S1: Bacterial load (CFU/ml) of mature MRSA biofilms adherent to various implant material disks: **(A)** without treatment (- CTRL); after 24 hour exposure to **(B)** 1-; **(C)** 5- and **(D)** 10-times the MBC of rifampicin and ciprofloxacin. Results are shown as individual values (N=17), horizontal lines indicate means and error bars indicate 95% CI. CFU: Colony forming units; MBC: Minimal bactericidal concentration;
