# Supplementary material for: Influence of surface characteristics of implant materials on MRSA biofilm formation and effects of antimicrobial treatment
Source: Front Microbiol. 2023 Apr 20;14:1145210. doi: 10.3389/fmicb.2023.1145210 (PMC10159048; doi:10.3389/fmicb.2023.1145210)
Supplement: Supplementary file 2 [file Image_2.pdf]

Supplementary Material

Influence of surface characteristics of implant materials on MRSA biofilm formation and effects of antimicrobial treatment

Authors:
S.C.J. van Dun[1*], M. Verheul[1], B.G.C.W. Pijls[2], J. van Prehn[3], H. Scheper[1], F. Galli[4], P.H. Nibbering[1], M.G.J. de Boer[1]

*__Corresponding author:__
S.C.J. van Dun
Department of Infectious Diseases, Room E5-07
Address: Leiden University Medical Center, Albinusdreef 2, 2300 RC Leiden, the Netherlands
Tel: +31715261779
Email: s.c.j.van_dun@lumc.nl

2. Implant-associated material disks

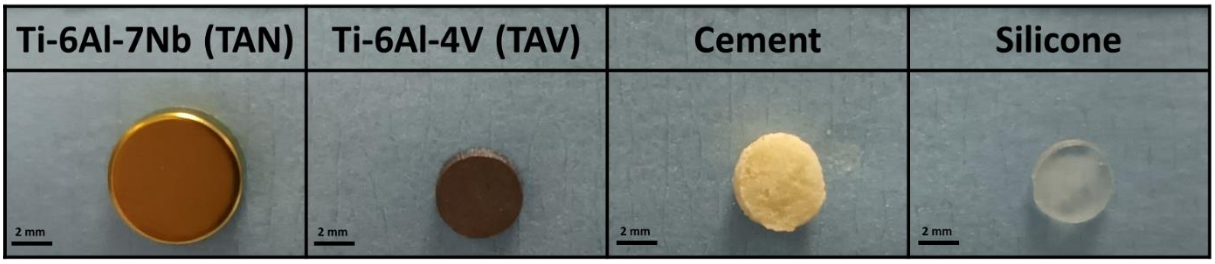

Supplementary Figure S2: Disks made out of implant-associated materials, with from left to right: Ti-6Al-7Nb (TAN); Ti-6Al-4V (TAV); Orthopedic bone cement and silicone. All materials are to scale, with the scale bar indicating 2cm.
