# Supplementary material for: Influence of surface characteristics of implant materials on MRSA biofilm formation and effects of antimicrobial treatment
Source: Front Microbiol. 2023 Apr 20;14:1145210. doi: 10.3389/fmicb.2023.1145210 (PMC10159048; doi:10.3389/fmicb.2023.1145210)
Supplement: Supplementary file 3 [file Table_1.docx]

***Supplementary Material***

**Influence of surface characteristics of implant materials on MRSA biofilm formation and effects of antimicrobial treatment**

**Authors:**

S.C.J. van Dun^1*^, M. Verheul^1^, B.G.C.W. Pijls^2^, J. van Prehn^3^, H. Scheper^1^, F. Galli^4^, P.H. Nibbering^1^, M.G.J. de Boer^1^

***Corresponding author:**

S.C.J. van Dun

Department of Infectious Diseases, Room E5-07

Address: Leiden University Medical Center, Albinusdreef 2, 2300 RC Leiden, the Netherlands

Tel: +31715261779

Email: s.c.j.van_dun@lumc.nl

1. **Surface parameters of implant materials with different maturities of staphylococcal biofilms**

**Supplementary Table S1:** Surface parameters of the implant materials Ti-6Al-7Nb (TAN) Ti-6Al-4V (TAV), orthopedic bone cement and silicone. Surface roughness Ra (nm) and peak-to-valley roughness Rt (nm) of implant material disks without biofilm (-), with 24 h biofilm and with 7 d biofilm. Surface parameters were determined from 100x100 μm AFM images. Values are mean with 95% confidence interval (CI) in nm.

| Material | Biofilm | Mean Ra (95% CI) (nm) | Mean Rt (95% CI) (nm) |
| --- | --- | --- | --- |
| Ti-6Al-7Nb (TAN) | - | 187 (152-222) | 1883 (1710-2055) |
|  | 24 h | 212 (190-234) | 2005 (1818-2192) |
|  | 7 d | 186 (160-211) | 2054 (1883-2226) |
| Grade 5 Ti-6Al-4V (TAV) | - | 279 (236-322) | 2313 (2060-2566) |
|  | 24 h | 268 (220-316) | 2452 (2138-2767) |
|  | 7 d | 270 (244-296) | 2363 (2134-2591) |
| Orthopedic bone cement | - | 1379 (1241-1517) | 7354 (6642-8067) |
|  | 24 h | 1117 (904-1329) | 6763 (6126-7400) |
|  | 7 d | 1551 (1412-1690) | 8479 (7722-9236) |
| Silicone | - | 623 (439-815) | 4929 (4340-5517) |
|  | 24 h | 623 (465-781) | 4683 (3872-5493) |
|  | 7 d | 636 (495-778) | 5214 (4133-6295) |
